# Cervical dilation assessment in simulators compared to a visual tool: A randomized study

**DOI:** 10.1590/1518-8345.6102.3882

**Published:** 2023-03-27

**Authors:** Natalucia Matos Araújo, Angela Meguni Ochiai, Joyce da Costa Silveira de Camargo, Edson Yassushi Ussame, Ruth Hitomi Osava, Lucia Cristina Florentino Pereira da Silva

**Affiliations:** 1 Universdade de São Paulo, Escola de Artes, Ciências e Humanidades, São Paulo, SP, Brasil; 2 Universidade de São Paulo, Hospital Universitário, São Paulo, SP, Brasil

**Keywords:** Cervix Uteri, Simulation Training, Teaching, Labor, Obstetric, Obstetric Nursing, Obstetrics, Cuello del Útero, Entrenamiento Simulado, Enseñanza, Trabajo de Parto, Enfermería Obstétrica, Obstetricia, Colo do Útero, Treinamento por Simulação, Ensino, Trabalho de Parto, Enfermagem Obstétrica, Obstetrícia

## Abstract

**Objective:**

to verify the correct assessment rate when using direct visual comparison in the cervical dilation measures in hard-consistency cervix simulation models.

**Method:**

an open-label and randomized study conducted with 63 Obstetrics students that were designated either to use direct visual comparison in a dilation guide or not. The students estimated cervical dilation blindly in simulators with different dilations. The primary outcome was the correct assessment rate.

**Results:**

the students performed 141 tests. A higher correct assessment rate was found in the Experimental Group than in the Control Group (47.3% versus 27.2%; p<0.001; Odds Ratio = 2.41; 95% Confidence Interval = 1.62-3.58).

**Conclusion:**

the direct visual comparison increased precision of the cervical dilation assessment in cervix simulation models, with the possibility of being beneficial in laboratory training. Brazilian Registry of Clinical Trials No. U1111-1210-2389.

## Abstract (es)

**Objetivo:**

comprobar la tasa de evaluación correcta mediante la comparación visual directa de las medidas de dilatación cervical en modelos de cuello uterino de consistencia dura.

**Método:**

estudio aleatorizado abierto con 63 estudiantes de obstetricia a los que se les asignó usar o no la comparación visual directa con una guía de dilatación. Los estudiantes estimaron de forma ciega la dilatación cervical en simuladores con diferentes dilataciones. El resultado primario fue la tasa de evaluación correcta.

**Resultados:**

los estudiantes realizaron 441 pruebas. Se observó una mayor tasa de evaluación correcta en el grupo experimental que en el grupo control (47,3% versus 27,2%; p < 0,001; Odds Ratio = 2,41; intervalo de confianza del 95% = 1,62-3, 58).

**Conclusión:**

la comparación visual directa aumentó la precisión de la evaluación de la dilatación cervical en modelos de simulación de cuello, lo que podría ser beneficioso en el entrenamiento de laboratorio. Registro Brasileño de Ensayos Clínicos n.º U1111-1210-2389.

## Abstract (pt)

**Objetivo:**

verificar a taxa de avaliação correta com o uso da comparação visual direta nas medidas de dilatação cervical em modelos de simulação de colo com consistência dura.

**Método:**

estudo randomizado aberto com 63 estudantes de obstetrícia que foram designados para usar comparação visual direta em um guia de dilatação ou não. Os estudantes estimaram cegamente a dilatação cervical em simuladores com diferentes dilatações. O desfecho primário foi a taxa de avaliação correta.

**Resultados:**

os estudantes realizaram 441 testes. Foi encontrada maior taxa de avaliação correta no grupo experimental do que no grupo controle (47,3% versus 27,2%; p <0,001; Odds Ratio = 2,41; intervalo de confiança de 95% = 1,62-3,58).

**Conclusão:**

a comparação visual direta aumentou a precisão da avaliação da dilatação cervical em modelos de simulação de colo, podendo ser benéfica no treinamento em laboratório. Registro Brasileiro de Ensaios Clínicos nº U1111-1210-2389.

**Highlights**

(1) Direct visual validation increases precision of cervical dilation assessments.

(2) Direct visual validation is useful in initial and advanced cervical dilations.

(3) A simple measuring tool can be employed in direct visual validation.

## Introduction

Practical skills training among undergraduate students is challenging, considering suitable learning and respectful care to women during delivery. Among the delivery practices, cervical dilation assessment through vaginal examination, in which progress of labor is verified, is important in clinical decision-making; an incorrect diagnosis can cause physical and emotional harms to parturients ^(1)^ . 

In a traditional obstetric environment, the students need to perform several vaginal examinations in order to develop proprioceptive skills to assess cervical dilation. Generally, these exams are performed under the supervision of an experienced professional, who then repeats it with each woman. This repetition is a sensitive action, as women generally consider this procedure as invasive and often relate it to feelings of shame, constraint, fear or discomfort. In addition to that, it can increase the infection risk ^(1- 2)^ . 

However, being capable of performing a correct assessment of cervical dilation via a digital examination is recognized as an essential skill in the management of delivery pain ^(1- 3)^ . 

In this way, the clinical practice with real parturients to train the students in the development of digital examination skills becomes dichotomous: on the one hand, the invasive technique of vaginal examination should be minimized and, on the other hand, the objective should be that apprentices with few opportunities have sufficient competence in cervical dilation assessment. The challenge is set out and it is necessary to search for solutions.

A number of studies evidence that training in simulators increases learning retention and minimizes the gap between knowledge about a procedure and its practical application ^(4- 5)^ . In Obstetrics, with regard to evolution of labor, perineal manikins have been used to teach cervical modifications (dilation, effacement, consistency and position), fetal presentation and position in relation to the maternal pelvis ^(4- 10)^ . These manikins are generally developed to achieve a realistic simulation, including soft cervical consistency. On the other hand, hard- consistency cervix models allow for better results among students in initial stages ^(7)^ . 

The students acquire the skill to perform a vaginal examination by means of a proprioceptive experience. However, they have difficulty assessing cervical dilation through the distance between the fingertips. They probably need something else than proprioceptive perception to improve their simulation learning results ^(11)^ . 

In order to assist the undergraduate Obstetric students in assessing cervical dilation in the laboratory, a tool called Cervical Dilation Guide (CDG) was created. The hypothesis is that using direct visual comparison increases accuracy of the results in the cervical dilation assessment.

Thus, the objective of this study was to verify the correct assessment rate when using direct visual comparison in the cervical dilation measures in hard-consistency cervix simulation models.

## Method

### Study design

This is an open-label and randomized study, conducted to evaluate the effect of direct visual comparison on Obstetrics students’ correct assessment rate using cervical dilation simulators without prior training in simulating vaginal examinations in simulation models.

### Locus

The study was conducted in the laboratory of a public university from the city of São Paulo, SP, Brazil. The laboratory is sized 21 x 5 meters, for a total of 105 square meters consisting in granite benches where the simulation models were arranged for the study.

### Period

The research was developed between August 22 ^nd^, 2018, and May 17 ^th^, 2019. 

### Population

The study population consisted of students regularly enrolled in the Obstetrics undergraduate course of a public university from São Paulo.

### Selection criteria

Second- and fourth-year Obstetrics students with no prior training in vaginal touch in simulation models, enrolled in the academic disciplines taught by the researchers of this study, were eligible for inclusion in the research and recruited in the classrooms due to ease of access, opportunity for presenting the research and scheduling the date and time for data collection in the laboratory, for those who voluntarily agreed to participate.

It is noted that vaginal touch simulation is not included in the list of academic disciplines targeted at the study population.

The exclusion criterion corresponded to students that took part in the tests in the dilation models to compare the soft and hard consistency cervices.

### Definition of the sample

A pilot study was conducted with 44 students for sample calculation, with the correct assessment rate as primary outcome. In this pilot group, the correct assessment rates were 24% in the Control Group and 58% in the CDG Group, with 80% statistical power and 5% alpha error. Therefore, sample size was calculated at 32 participants for each group: Cervical Dilation Guide (CDG) and Control, totaling 64 subjects.

### Study variables

The dependent variables were cervical dilation in the simulation models and the CDG measures.

The independent variables were age in full years, biological designation as male or female, the academic period enrolled in the Obstetrics course, the number of vaginal examinations performed, and the number of deliveries assisted during undergraduation.

### Instruments used for data collection

The instrument used for data collection consisted of a form prepared for this research divided into two sections: the first one contained data referring to the independent variables: age, gender, year of the undergraduate course in progress, number of vaginal examinations performed, and number of deliveries assisted during undergraduation. The second section comprised data corresponding to the dependent variables: measures verified by the students in all seven simulation models, and the measures they cited regarding direct visual comparison in the CDG.

### Data collection

We developed seven simulation models in our laboratory, externally consisting in a foam vulva coated with rubber, measuring 15 cm in height, 13 cm in width and 13 cm in depth; behind it there was an opening through which the cervix was fitted with the dilation chosen for the study and, so that there was no displacement during the assessments made by the participants, a sphere made of polyurethane foam measuring 26 cm in diameter, representing the pole cephalic, was used to fix the dilation. Internally, the vaginal canal was 4 cm long, through which the index and middle fingers were extended to achieve dilation.

Previously, soft-consistency cervix models were built, as they were more realistic; they had plastisol in their composition. However, after tests carried out by more experienced students in vaginal examination, a decrease in the correct assessment rate was observed, especially in dilations with larger diameters (>6 cm). Consequently, it was decided to make hard-consistency models made of rubber, silicone and polyurethane, thus improving the students’ perception. It is worth noting that this study will be published at a later time.

The authors ^(7)^ that tested the consistency of cervical models in obtaining more accurate results when examined by professionals with different levels of experience concluded that hard-consistency cervical models were better for beginners. 

Seven identical models with different cervical dilations were prepared to avoid external identification of each dilation. Thus, extreme and median cervical dilation measures were chosen for inclusion in the study (1.5 cm, 2.0 cm, 4.0 cm, 5.0 cm, 6.0 cm, 7.0 cm and 8.0 cm).

**Figure 1:**
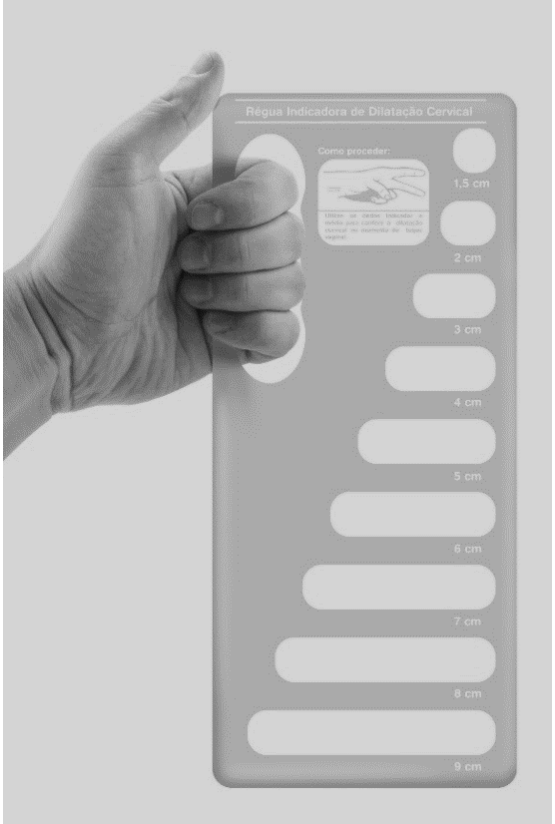
Cervical Dilation Guide (CDG)

For direct visual comparison, the CDG was used, an instrument for objective length measurement (ruler) made of transparent acrylic material, with a surface measuring 25 cm × 11 cm × 3 mm (height, width and thickness) and containing nine hollow geometric figures, oriented by diameter in the horizontal direction, corresponding to cervical dilations ranging from 1.5 cm to 9.0 cm (Figure 1). The CDG was registered by the University at the National Institute of Industrial Property (BR 302014004714-0). 

### Interventionist procedures

The main researcher generated a randomization sequence in size 4 permuted blocks in a 1:1 ratio. The students were drawn by using closed envelopes, and allocated to the control or intervention (CDG) groups.

Models with different dilations were placed in a random sequence on the laboratory bench before the procedure; in addition to that, the researchers and participants were blinded to cervical dilation. Individually, each student entered the laboratory room and was monitored by one of the researchers.

In each model, the students were instructed to use their dominant hand and introduce their index and middle fingers through the vagina up to the cervix. The fingers were then opened until their external tips reached the opposite margins of the cervical orifice. The distance was considered from the beginning of the outer tip of the index finger to the end of the outer tip of the middle finger. There was no time limit to perform the assessments. Thus, when assessing the dilation contained in the dilation models, the Control Group participants conveyed their estimates in centimeters, which were then simultaneously recorded on the form by one of the researchers. Likewise, the CDG Group participants initially assessed each dilation available in the dilation models, and then compared their estimates in the CDG that was presented by the researcher. At each confirmation, the researcher made the due records on the form.

At the end of the verification done by the participant, an external evaluator provided the actual measurements of cervical dilation found below each simulation model, and the researcher completed the second section of the data collection form.

### Statistical analysis

Mean/Standard Deviation were used to analyze the continuous parametric variables and median/interquartile range were employed for non-parametric distribution according to the Kolmogorov-Smirnov test. For the categorical variables, numbers and frequencies were used as percentages, and the chi-square test was employed to assess the association between the CDG and control groups. A p-value of 5% was defined as statistically significant. A logistic regression was performed to determine the Odds Ratio of the correct assessment rate between the CDG and control groups and construct a two-tailed 95% Confidence Interval (CI).

### Ethical aspects

The study followed the recommendations set forth in Resolution No. 466, dated December 12 ^th^, 2012, and the participants consented to their participation in writing by signing the Free and Informed Consent Form. 

The study was approved by the Committee of Ethics in Research with Human Beings of a public university under opinion No. 1,322,956 and Certificate of Presentation of Ethical Appreciation No. 49827815.2.0000.5390; approval was also obtained in the Brazilian Registry of Clinical Trials under No. U1111-1210 -2389, which can be accessed at https://ensaiosclinicos.gov.br/rg/RBR-243frc.

The manuscript was prepared based on the rules set forth in the Consolidated Standards of Reporting Trials (CONSORT).

## Results

**Figure 2:**
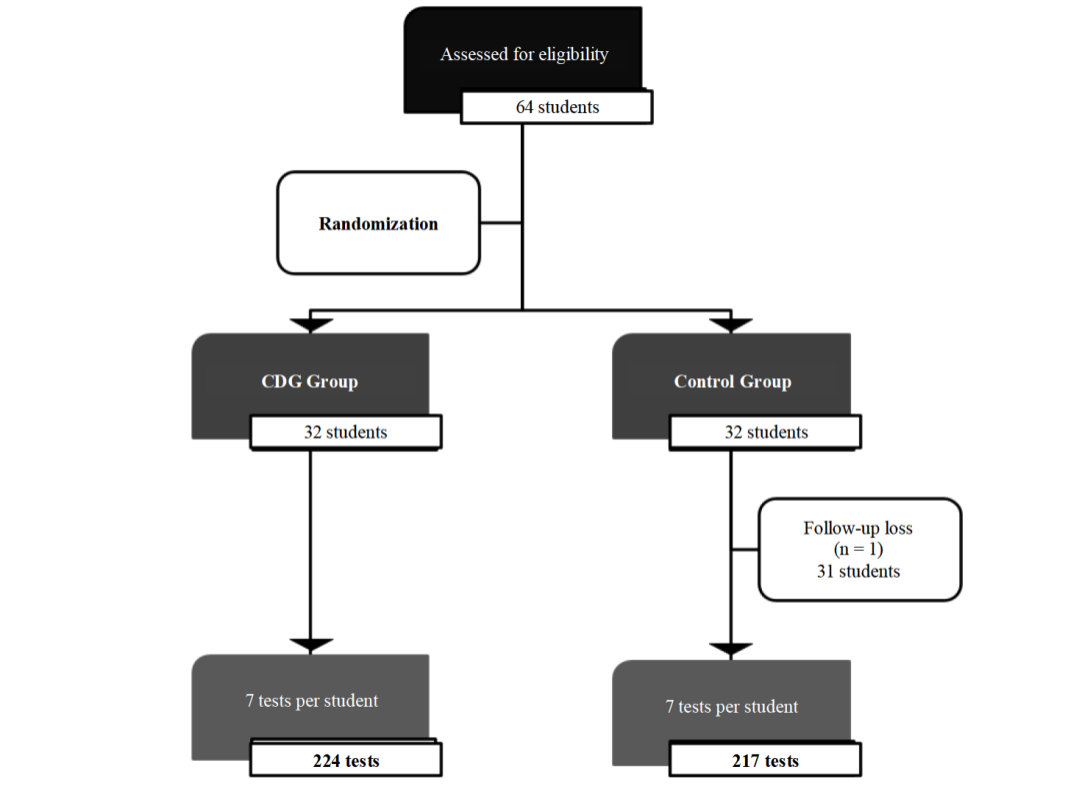
Recruitment and randomization

The recruitment and randomization diagram corresponding to the study is presented in Figure 2. 

*CDG = Cervical Dilation Guide

The characterization of the groups is reported in Table 1. 

**Table 1:** Characterization of the groups (n = 63). São Paulo, SP, Brazil, 2019

Characterization	CDG^*^ Group (32 students)	Control Group (31 students)
**Age Mean (Standard Deviation)**	21.6 (2.6)	21.9 (1.7)
**2^nd^ undergraduate year n(%)**	13 (40.6%)	12 (38.7%)
**4^th^ undergraduate year n(%)**	19 (59.4%)	19 (61.3%)
**Experience with vaginal examinations (≤ 3), n(%)**	21 (65.6%)	20 (64.5%)
**Normal delivery care (≤ 1), n(%)**	20 (62.5%)	22 (74.2%)

* CDG = Cervical Dilation Guide

A total of 64 students were included and randomly assigned to the CDG (n = 32) and control (n = 32) groups; one participant from the Control Group was discontinued from the study (Figure 2) because some data referring to the measurements of the dilations found in the models were not recorded in the form. Most of the participants were female (93.8%) and each student performed seven cervical dilation tests (1.5 cm, 2.0 cm, 

4.0 cm, 5.0 cm, 6.0 cm, 7.0 cm, and 8.0 cm); therefore, 441 tests were made.

**Figure 3:**
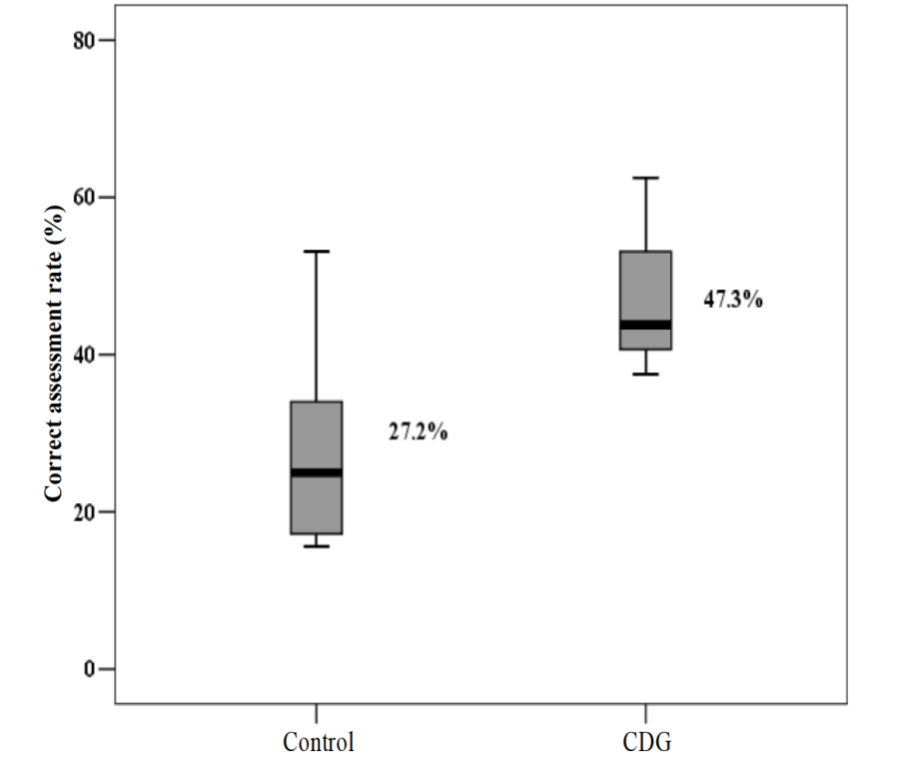
Correct assessment rate according to the groups (n = 441)

All 441 tests were divided (for the comparison) according to the groups: CDG (n = 224) and Control (n = 217). When the groups were compared for each cervical dilation, a significant difference was found for 1.5 cm, 7.0 cm and 8.0 cm (Table 2). However, considering the total tests, the rate of correct assessments using the CDG was significantly higher (47.3% vs. 27.2%; Odds Ratio = 2.41; 95% CI: 1.62–3.58; p<0.001) (Figure 3). 

**Table 2:** Correct assessment rate according to the groups (n = 63). São Paulo, SP, Brazil, 2019

Cervical dilatation (cm)	CDG^*^ Group (32 students)	Control Group (31 students)	OR^†^	CI^‡^	p^§^
1.5	19 (59.4%)	10 (32.3%)	3.07	1.09-8.61	0.033
2.0	20 (62.5%)	16 (51.6%)	-	-	0.383
4.0	12 (37.5%)	8 (25.8%)	-	-	0.319
5.0	15 (46.9%)	10 (32.3%)	-	-	0.236
6.0	12 (37.5%)	5 (16.1%)	-	-	0.056
7.0	14 (43.8%)	5 (16.1%)	4.04	1.24-13.23	0.021
8.0	14 (43.8%)	5 (16.1%)	4.04	1.24-13.23	0.021
Total tests (7 dilations)	224	217	-	-	-
Total of correct assessments	106 (47.3%)	59 (27.2%)	2.41	1.62-3.58	<0.001

* CDG = Cervical Dilation Guide;

† OR = Odds Ratio;

‡ CI = Confidence Interval;

§ p = Significance level

*CDG = Cervical Dilation Guide

## Discussion

The main finding of the current study was an increase in the Obstetric students’ success rate in cervical dilation assessment when using the CDG, when compared to hard-consistency cervix simulation models.

The accuracy of experienced midwives, nurses and physicians ranged from 49% to 58% in the simulation models ^(8, 9)^ . In this study, considering that the sample consisted of Obstetrics students with no previous training in cervical simulators and little or no experience in vaginal examinations, the rate of correct answers in the assessments was 47.3%. Using direct visual comparison increased the correct assessment rate by 2.4 times, which can be explained by the visualization of the space between the fingers when compared to equivalent measurements in centimeters in the CDG. 

When the groups were compared for each cervical dilation, a significant difference was found for 1.5 cm, 7.0 cm and 8.0 cm. Some authors report that, in medium dilations (5 cm-7 cm), accuracy decreases even among experienced professionals ^(10)^ , similarly to our findings. People are probably less accurate in assessing intermediate dilations than extreme ones; therefore, the dilations that did not show differences in our study should not be related to use of the CDG. 

Sophisticated simulations such as high-technology or virtual reality models have been emphasized in the literature. However, low-fidelity simulations remain effective for students to learn how to perform a vaginal examination during delivery, as these models allow for a repetitive practice, in addition to being simple and accessible ^(4- 5, 8- 9, 12)^ . 

A research study on cervical assessment simulation training ^(9- 10)^ shows that medical students tend to overestimate cervical dilations. After training, they found more success rates for smaller and median dilations when compared to larger ones. 

A number of studies recommend hard-consistency cervix manikin models to teach beginners ^(7)^ . Soft- consistency cervical models are more realistic simulators in delivery environments; however, the correct cervical dilation assessment rate was 19% when compared to 54% in the hard-consistency cervix models ^(7)^ . 

As cervical alterations are not only limited to dilation, some authors ^(7)^ state that students can benefit from learning dilation in rigid models before advancing to soft-consistency ones, with which they can experiment other cervical parameters, such as position, consistency and effacement. 

Cervical assessment are useful to identify the evolution of dilation and when it is complete, it predicts delivery ^(10)^ ; however, in real life, the exact measurement of dilation often plays a secondary role, as other clinical observations are combined in assessing progression of labor, even so, training students in the acquisition of this skill is crucial, as the standard assessment for cervical dilation, although subjective, remains the traditional digital examination ^(2)^ . 

In this study, the CDG was developed as a low-cost and practical measuring tool that uses the diameter as a reference. It is simple, easy to use, affordable and requires no prior training.

The key point of this tool is resorting to a visual objective measurement as a complement to finger proprioception. This learning process can be achieved with a simple ruler or measuring tape, which are widely available, easy to reproduce, versatile and cost-effective ^(12)^ . The students can benefit from adding visual perception ^(11)^ . 

Other strategies have been used to improve this learning process. For example, the students measure the distance between the index and middle fingers to determine the actual width of that space ^(9)^ ; another way is to have previous visual and tactile contact with cervical dilations in manikin models ^(11)^ . 

In this study, direct visual comparison improved the students’ performance in cervical dilation assessment in the simulation models, with the possibility of being incorporated into laboratory training of labor, promoting a more careful teaching-learning process, as the students will be able to repeat their assessments as many times as necessary, minimizing repetitive vaginal touches in women to acquire this skill.

This study is limited by the fact that the CDG was designed for direct visual comparison, thus only evaluating cervical dilation. Other important variables such as effacement, consistency and position were not included. The cervix simulation models and the CDG were tested for our needs; however, they have not been validated by experts. For greater accuracy of the assessments, the participants could have performed at least three assessments on each dilation model, comprising intra-rater reliability.

From the results shown, statistically significant in the comparison of the correct assessment rate between the experimental and control groups (47.3% versus 27.2%), it can be stated that the direct visual comparison as an educational support tool is capable of assisting students in cervical dilation assessment in uterine cervix simulators. To the best of our knowledge, this is the first study to use visual comparison in addition to proprioception skills to train students in simulation models, which will certainly contribute to advancement of knowledge and of the clinical practice, as well as of patient safety in the Obstetrics area.

## Conclusion

Using direct visual comparison increased precision of the cervical dilation assessment in hard-consistency cervix simulation models, with the possibility of being beneficial in laboratory training. More research studies are needed to compare the students’ performance by combining simulation training and clinical performance in delivery settings.
